# Bone marrow stroma cells derived from mononuclear cells at diagnosis as a source of germline control DNA for determination of somatic mutations in acute myeloid leukemia

**DOI:** 10.1038/bcj.2017.93

**Published:** 2017-10-06

**Authors:** H Mujahed, M Jansson, S Bengtzén, S Lehamnn

**Affiliations:** 1 Department of Medicine, Huddinge, Center for Haematology and Regenerative Medicine, Karolinska Institute, Stockholm, Sweden; 2 Department of Medical Sciences, Haematology, Uppsala University, Uppsala, Sweden

The introduction of next generation sequencing (NGS) techniques has revolutionized the genetic characterization of malignant diseases.^1^ Not only has NGS techniques lead to new research tools opening for ground breaking discoveries but the techniques are now being introduced in clinical routine, which will transform the way malignant diseases are characterized and diagnosed. However, optimal determination of somatic mutations in patient samples requires not only tumor DNA but also a germline control DNA. The importance of germline mutations in acute myeloid leukemia (AML) has recently come into special focus as being an integrated part in the new WHO classification.^2^ Thus, somatic mutations must be clearly distinguished from germline mutations and germline genetic variants and there is an increasing need for a good source of germline DNA when defining mutations in AML in the routine care. In research, a significant problem is that the only material that is available from an AML patient may be a biobanked vitally frozen vial taken at the diagnosis. Finding a reliable source of germline DNA is then crucial in research as well as for clinical routine. Several approaches to attain germline DNA in AML are used and have been proposed. Still, many of these approaches imply different limitations where skin biopsies and buccal swabs/washes can be hampered by contamination of leukemic cells^3^ as well as low amounts of DNA and the use of T cells is limited by the presence of evolutionary early somatic mutations like *DNMT3A* mutations that also are present in the malignant clone.^4^ Also, these approaches require additional sampling that has to be implemented in the routine. Remission samples are not possible to obtain for all patients as not all will achieve a complete remission and as pre-leukemic mutations may also be present in remission. Motivated by these problems, we aimed to find a reliable method to get out germline DNA from the same sample as from which the AML cells at diagnosis had been obtained. For this purpose, we choose to focus on the non-hematopoietic cell population present in the diagnostic AML bone morrow sample and hypothesized that bone marrow derived stroma cells (BMS) would fit the purpose. Our aim was also to find substantial amounts of germline DNA from each patient without contamination of leukemic cells and that this DNA could be used as a germline control for mutations found in the AML cells.

With this aim, we thawed, seeded and growed adherent cells from vitally frozen, Ficoll separated, bone marrow aspirates taken at the time of diagnosis of AML. The purpose was to eliminate leukemic cells and in parallel, obtain up to 5 μg of germline genomic DNA from non-malignant BMS. Forty-eight hours after seeding, unattached cells were washed away while BMS was attached to the bottom of the flask and were monitored by light microscopy for their ability to form colonies and retain a flattened shape. The culture was maintained in MyeloCult with 10% FBS for the first 2 weeks then in DMEM-GlutaMax and 10% FBS up to 6 weeks. For detailed description of culture procedure, Supplementary Material. At 2 and 6 weeks after seeding, cells were counted and characterized.

Six AML patients of which five had monosomy seven were chosen for the culture in order to facilitate identification of malignant cells in the BMS population. Patient characteristics are shown in Table 1. In addition to the routine karyotypic examination, the AML samples also underwent targeted exome sequencing of 32 AML genes in order to characterize the samples with regard to recurrent AML mutations and to exclude their presence in the cultured BMFs. Mutations in the AML cells were found in *DNMTT3A, EZH2, FLT3, IDH1, KRAS, NRAS, TET2, RUNX1, PTPN11, SF3B1, TP53* and *U2AF1* genes (Table 1).

Figure 1a shows representative growth curves of BMS from AML patients. Population doubling time (PDT) for BMSs varied from 8 to 16 days (mean 11 days) between patients. The average seeding density was between 1500 and 2500 cells cm^–2^. Frequent trypsinization and subculturing had a negative effect on BMS growth. Thus, we used a multi-layer culture flask and BMS cells were trypsinized after 2 and 4 weeks for the majority of the samples. BMSs were kept in culture for up to 6 weeks to obtain sufficient amount DNA (Figure 1a). The growth resulted in cells morphologically characterized by a fibroblast like morphology and relatively large nuclei (Figure 1b).

Flow cytometry was used to characterize primary AML cells and cultured BMS cells (Supplementary Methods). Results from flow cytometry analysis in AML blasts and BMSs in two representative AML patients are shown in Supplementary Table 1. The AML blasts expressed the leukocyte common antigen CD45, the stem cell markers CD34 and CD117, the myeloid marker CD13 as well as CD38 and HLA-DR. In addition, one AML sample also expressed the lymphoid markers CD7 and CD19 defining a biphenotypic AML. In contrast, BMSs were negative for CD45 and CD34 but positive for CD73, CD105 and CD90. Cultured BMSs from the biphenotypc AML lacked expression of lymphoid markers. This shows that *in vitro* culture of adherent cells from diagnostic AML samples cultured under these conditions expand a cell population that immunphenotypically could be characterized either as fibroblasts or mesenchymal stem cells (MSC).

In order to further characterize the potential of the cultured cells to differentiate into different cell types that typically can be derived from MSCs, we applied a differentiation culture procedure that is described in detail in Supplementary Material and in previously published studies.^5^ The differentiation assay reviled that the cultured BMS cells were able to differentiate into osteoblasts and adipocyte (Figure 1d) but not chondroblasts (data not shown). In contrast to what has been described for BM MSCs that lose their ability to differentiate into osteocytes after few passages,^6^ the cells in our culture retained their osteogenic differentiation potential also after 2 months in culture. Morphological presentation of cultured cells is shown in Figure 1.

To exclude malignant contamination of the BMS culture or presence of any of the genetic aberrations found in the AML samples in the cultured cells, genetic analysis was performed on cultured cells. A detailed description of the methods is presented in Supplementary Methods. To exclude monosomy 7 in the BMS cells, fluorescence *in situ* hybridization (FISH) was performed in all cells cultures and no cells with monosomy 7 could be detected in any of the cultures from any of the six patients (Figure 1c). For mutations in the AML cells, specific pyroprimers were designed to screen for mutations in *DNMTT3A, EZH2, FLT3, IDH1, KRAS, NRAS, TET2, RUNX1, PTPN11, SF3B1, TP53* and *U2AF1* in the BMS cells (Supplementary Table 2 and Supplementary Methods). The original pyrosequencing results are shown in Supplementary Figure 1. BMS cultures all showed wild-type variants for any of the genes that were mutated in the respective AML sample. These analyzes confirm the absence of AML-specific genetic aberrations in cultured BMS cells derived from the diagnostic AML sample and suggest that these cells could be used as a control DNA to confirm that genetic aberrations in the AML cells are somatic mutations.

Interestingly, genetic aberrations of BMS have previously been reported.^7, 8^ However, these mutations do not overlap with aberrations found in the malignant clone. The mechanisms for how these BMS specific aberrations occur or their impact on the malignant development remains unclear and needs to be subject to further studies. Nevertheless, BMS can still be used to exclude germline presence of genetic aberrations found in the AML clone.

The primary finding of growing bone marrow cells based on their ability to attach to plastic surfaces was described by Friedenstein and Castro-Malaspina.^9, 10^ As the bone marrow contains a heterogeneous cell population, later studies aimed to define subpopulations of BMS. Gronthos *et al.*^11^ presented the use of cell surface marker STRO-1^Bright^ VCAM-1^+^ to sort out a homogenous stroma population. However, our primary aim was to expand non-leukemic stroma cells and not a particular population of stroma cells. Now, we and others^12^ have shown that after few passages of BMS culture, non-adherent haematopoietic cells disappears either by washing away or by undergoing apoptosis resulting in a growth of a non-malignant and non-hematopoietic cell population.

In conclusion, our findings show that large amounts of non-hematopoietic cells defined as stroma cells can be derived from culturing adherent cells from mononuclear cells in the diagnostic bone marrow sample of AML patients. Furthermore, we show that these BMS cells can be used to exclude germline presence of mutations found in AML cells and thus define somatic mutations in AML patients. We suggest this can be a useful approach to obtain AML DNA as well as the control germline DNA from the same sample without additional sampling in the research setting as well as in clinical routine.

## Figures and Tables

**Figure 1:**
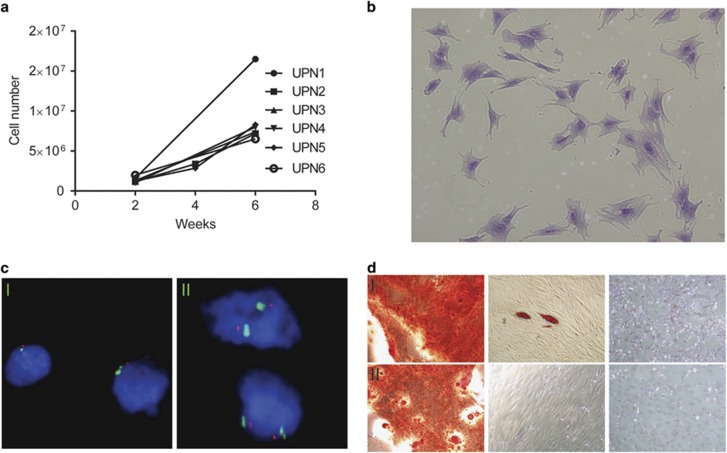
BMS growth and characterization. (**a**) BMS growth curves. (**b**) Crystal violet stain of stroma cell cultures (UPN2). (**c**) Fluorescence *in situ* hybridization (FISH) analysis of chromosome 7. Bone marrow cells from acute myeloid leukemia (AML) patients with monosomy 7 showing AML blasts (I) and cultured stroma cells (II). Probe against 7q31 show one red signal in AML cells and two signals in BMS cells. (**d**) Differentiation of BMS cells, left panels show osteogenic differentiation stained with alizarin red. Middle panels show adipogenic differentiation stained with Oil red O. Right panels show undifferentiated cells as negative controls. Both samples are from AML patients diagnosed with monosomy 7, BMS cells were in culture for 2 weeks (UPN5) top raw (I) or ten weeks (UPN2) bottom raw (II) prior to differentiation assay.

**Table 1:** Patient characteristics

*Patient*	*Age*	*Sex*	*Blast %*	*Diagnosis*	*Monosomy 7*	*Normal karyotype*	*Mutation*
UPN1	43	F	88	AML	No	Yes	*FLT3*,*IDH1*
UPN2	64	F	46	AML	Yes	No	*EZH2*,*FLT3*
UPN3	81	F	58	AML	Yes	No	*PTPN11*,*SF3B1*
UPN4	71	M	70	AML	Yes	No	*KRAS*,*TP53*,*U2AF1*
UPN5	51	F	50	s-AML	Yes	No	*DNMT3A*,*IDH1*,*NRAS*
UPN6	77	M	56	AML	Yes	No	*TET2*,*RUNX1*,*NRAS*

Abbreviations: AML, acute myeloid leukemia; F, female; M, male.
