# Supplementary material for: Bone marrow stroma cells derived from mononuclear cells at diagnosis as a source of germline control DNA for determination of somatic mutations in acute myeloid leukemia
Source: Blood Cancer J. 2017 Oct 6;7(10):e616–. doi: 10.1038/bcj.2017.93 (PMC5678220; doi:10.1038/bcj.2017.93)
Supplement: Supplementary Figure Legends [file bcj201793x1.docx]

**Figure legend, Supplementary Figures**

**Supplementary Figure 1.** Mutational analysis of AML blast cells and bone marrow stroma. Pyrograms from pyrosequencing analysis for different mutations present in AML blasts of all patients. In parallel, the same analyses were performed in bone marrow stroma cells.
