# Supplementary material for: Bone marrow stroma cells derived from mononuclear cells at diagnosis as a source of germline control DNA for determination of somatic mutations in acute myeloid leukemia
Source: Blood Cancer J. 2017 Oct 6;7(10):e616–. doi: 10.1038/bcj.2017.93 (PMC5678220; doi:10.1038/bcj.2017.93)

Supplementary figure 1.

Sample ID: UPN1 AML-blasts
Assay: IDH1_R132H

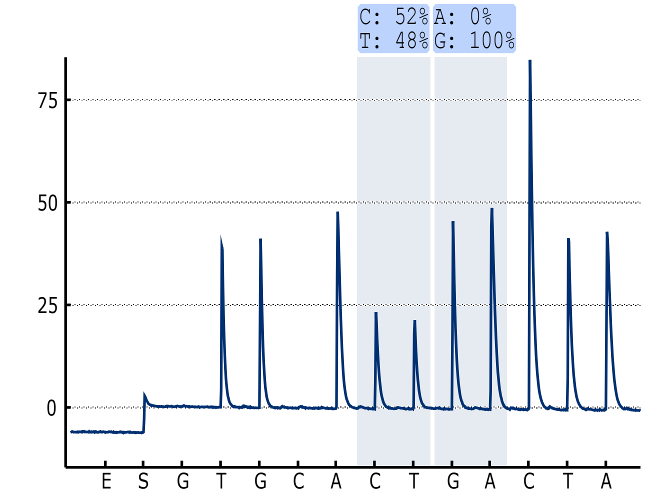

Sample ID: UPN1 BMS
Assay: IDH1_R132H

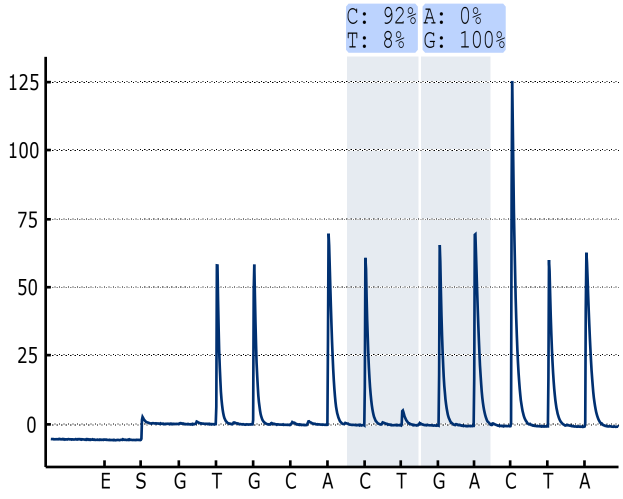

Sample ID: UPN1 AML-blasts
Assay: FLT3_g.28608217-28608217insC

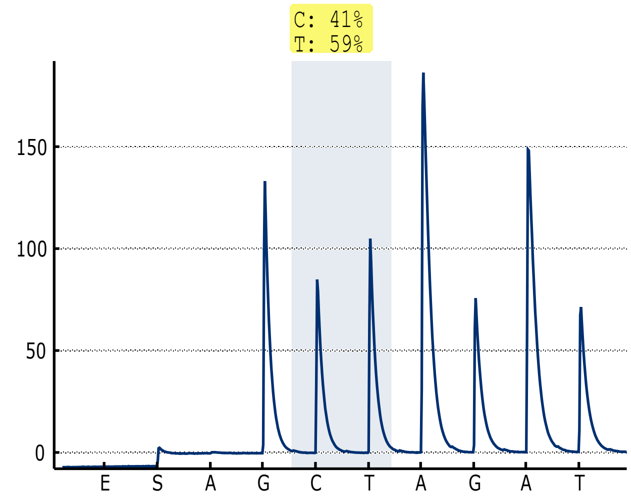

Sample ID: UPN1 BMS
Assay: FLT3_g.28608217-28608217insC

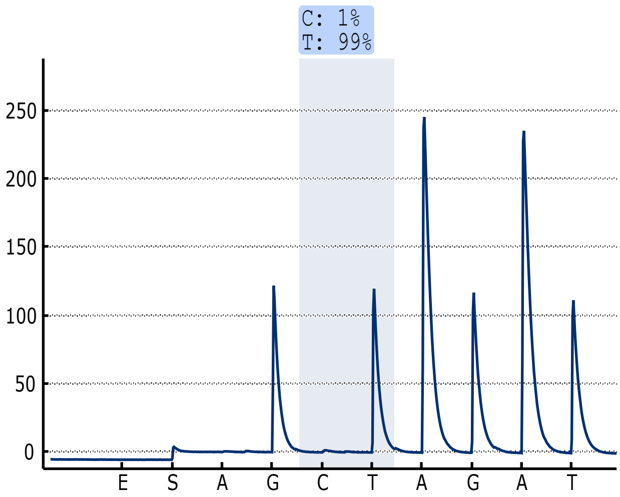

Sample ID: UPN2 AML-blasts
Assay: EZH2_E196*

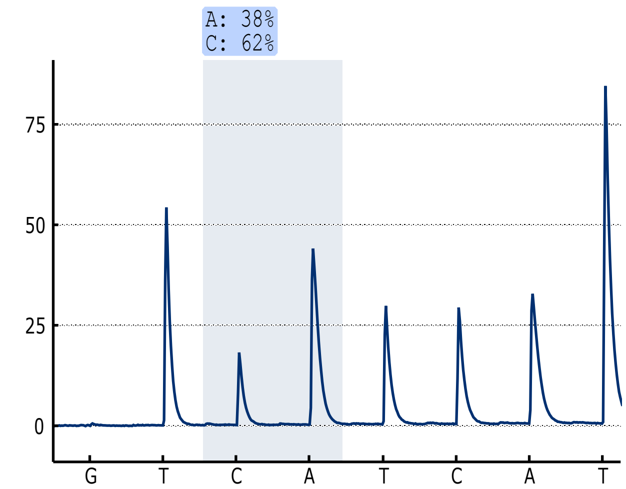

Sample ID: UPN2 BMS
Assay: EZH2_E196*

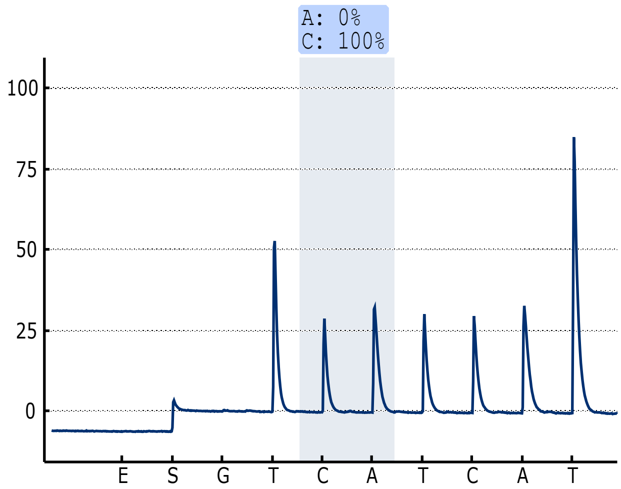

Sample ID: UPN2 AML-blasts
Assay: FLT3_D385Y

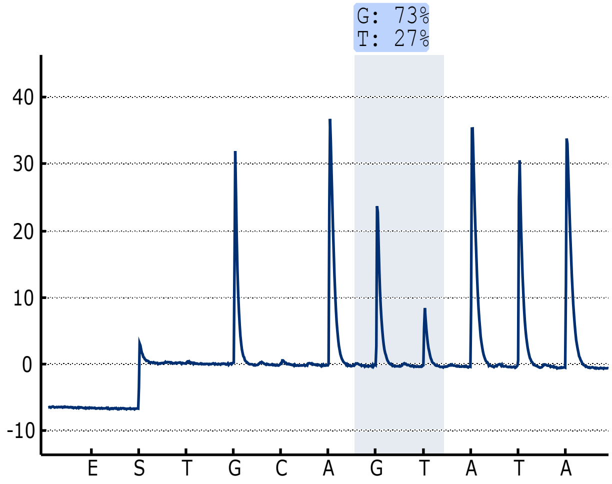

Sample ID: UPN2 BMS
Assay: FLT3_D385Y

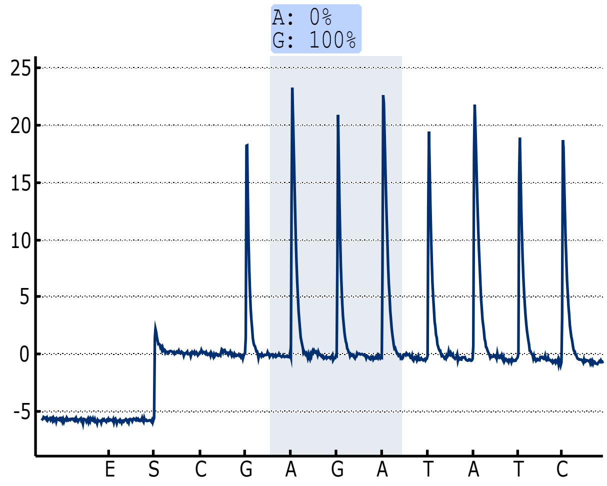

Sample ID: UPN3 AML-blasts
Assay: PEPN11_E76K

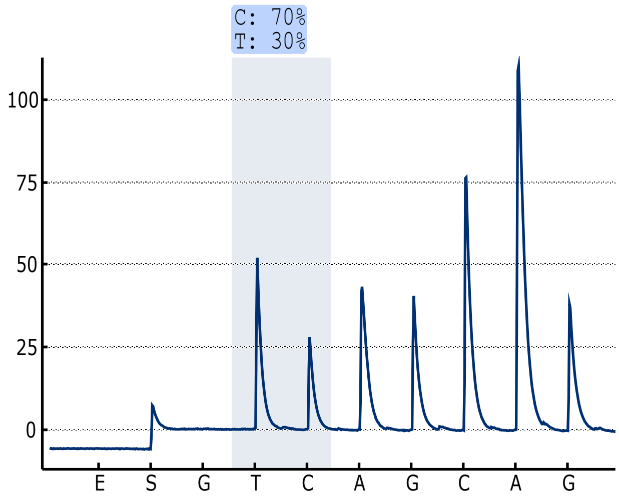

Sample ID: UPN3 BMS
Assay: PEPN11_E76K

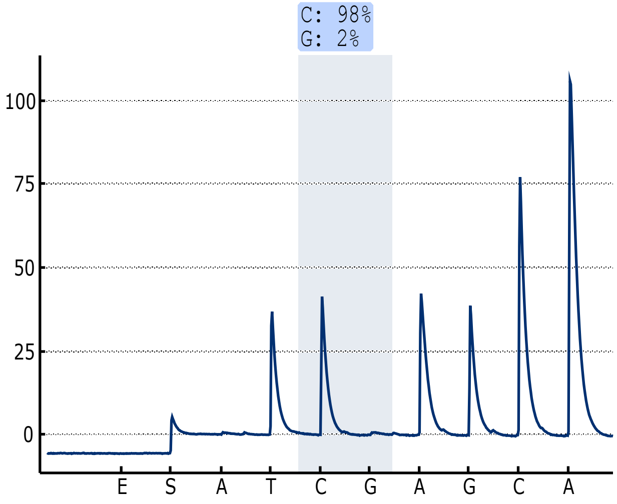

Sample ID: UPN3 AML-blasts
Assay: SF3B1_R625G

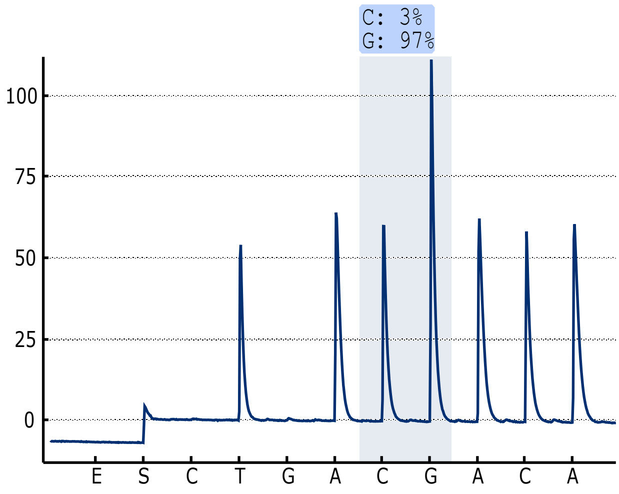

Sample ID: UPN3 BMS
Assay: SF3B1_R625G

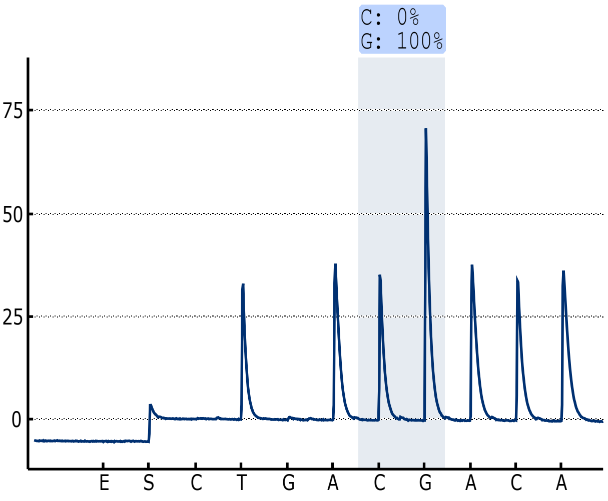

Sample ID: UPN4 AML-blasts
Assay: KRAS_G12V

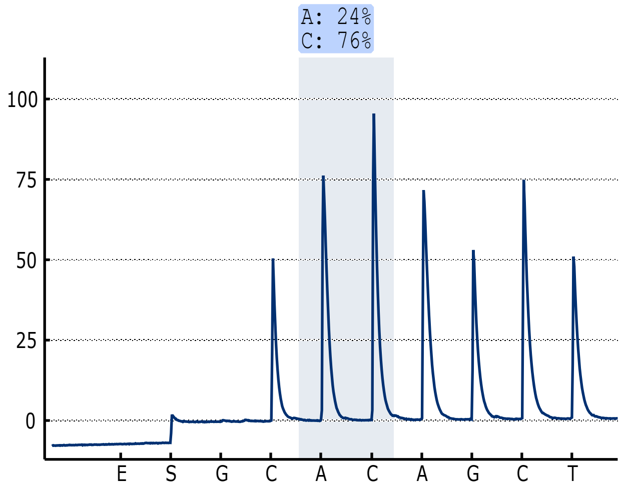

Sample ID: UPN4 BMS
Assay: KRAS_G12DV

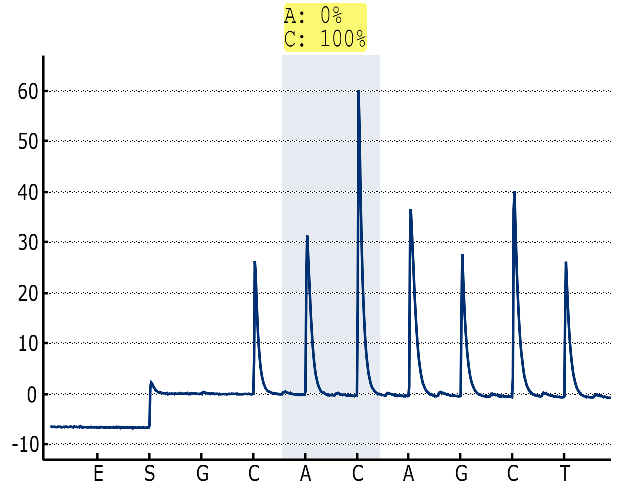

Sample ID: UPN4 AML-blasts
Assay: TP53_H193R

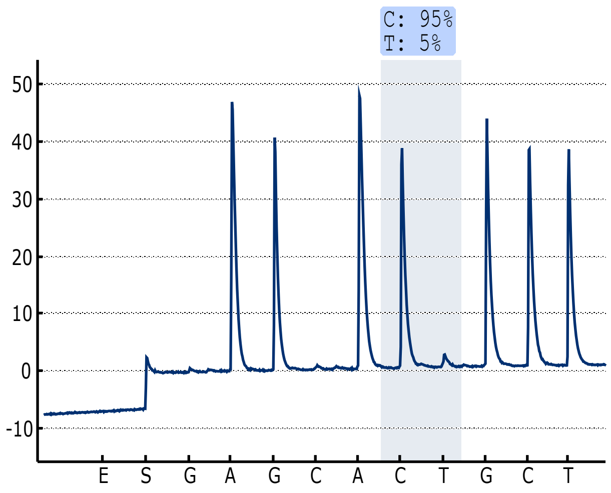

Sample ID: UPN4 BMS
Assay: TP53_H193R

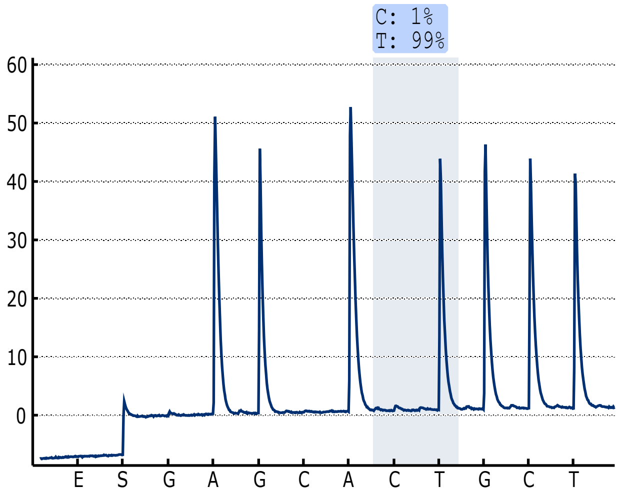

Sample ID: UPN5 AML-blasts
Assay: NRAS_G12D

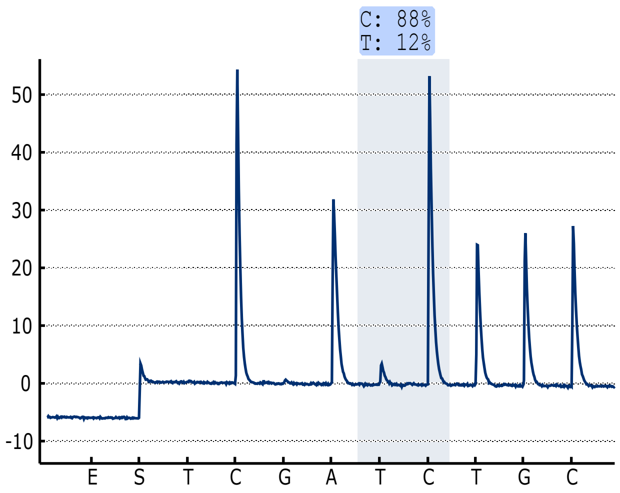

Sample ID: UPN5 BMS
Assay: NRAS_G12D

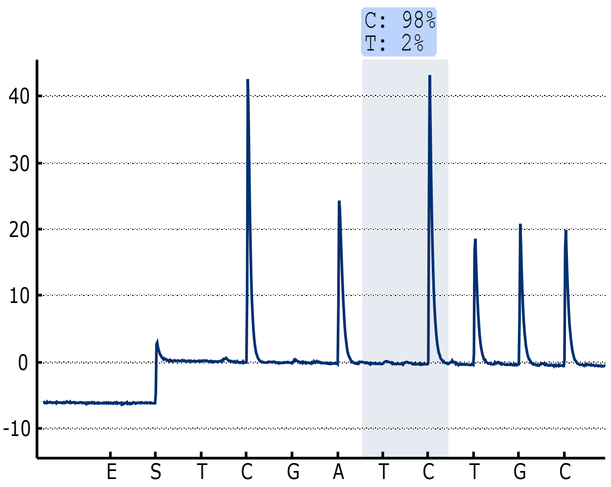

Sample ID: UPN5 AML-blasts
Assay: DNMT3A_R882H

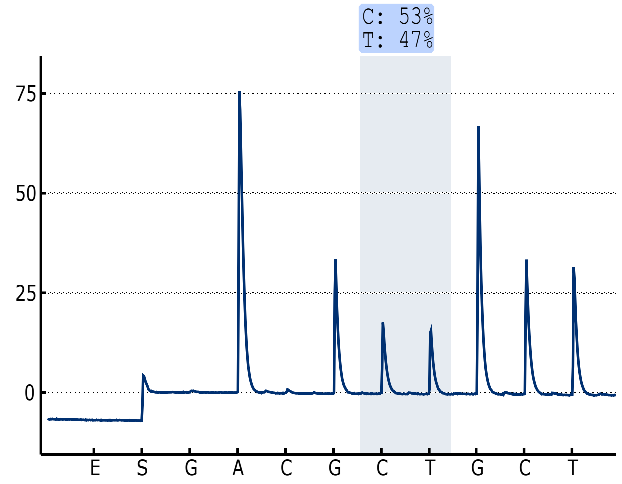

Sample ID: UPN5 BMS
Assay: DNMT3A_R882H

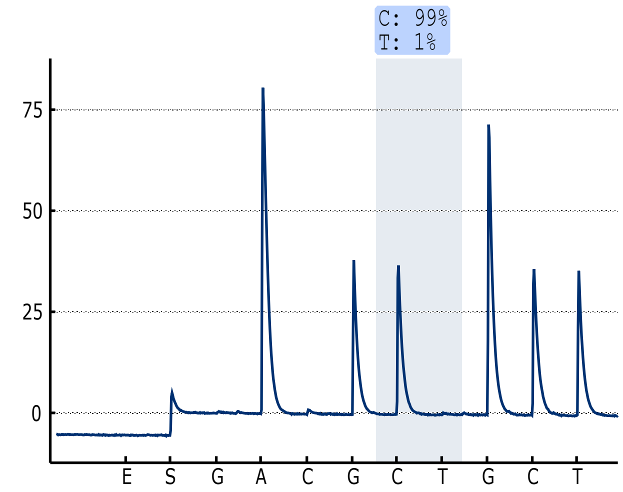

Sample ID: UPN6 AML-blasts
Assay: TET2_R1214W

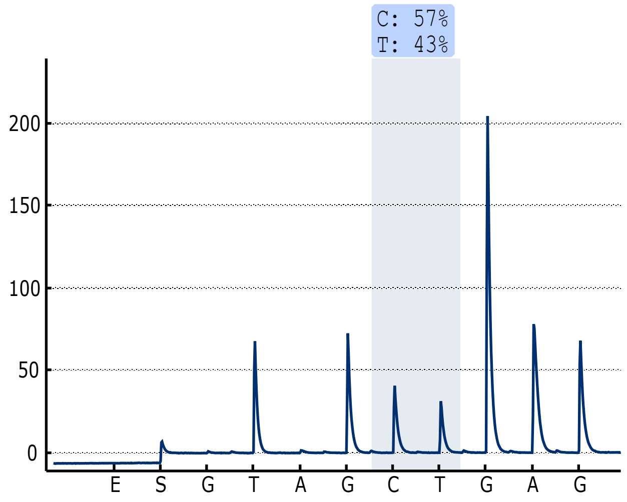

Sample ID: UPN6 BMS
Assay: TET2_R1214W

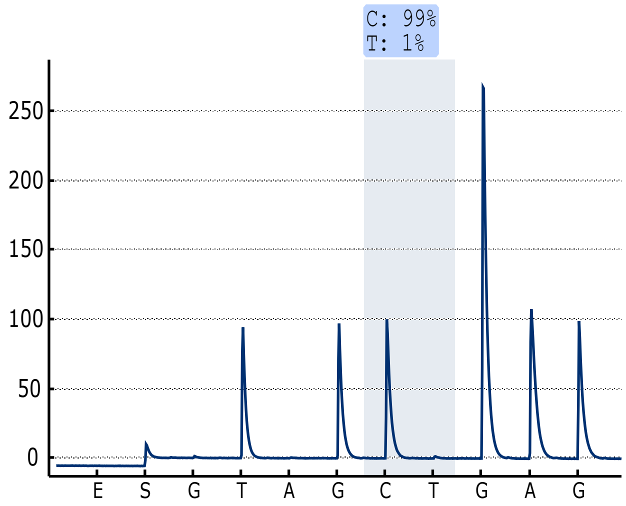

Sample ID: UPN6 AML-blasts
Assay: RUNX1_R166*

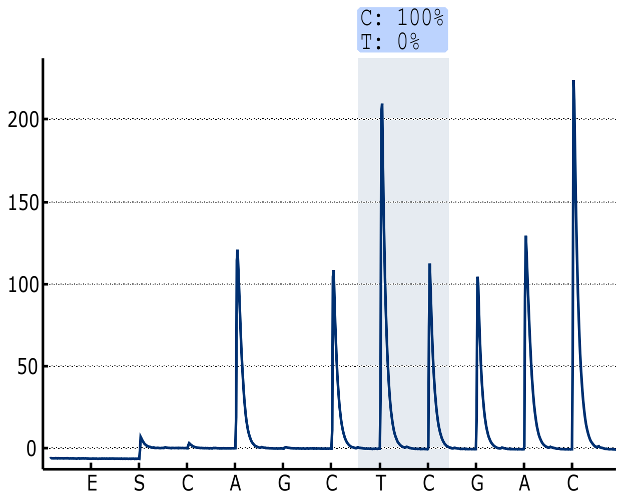

Sample ID: UPN6 BMS
Assay: RUNX1_R166*

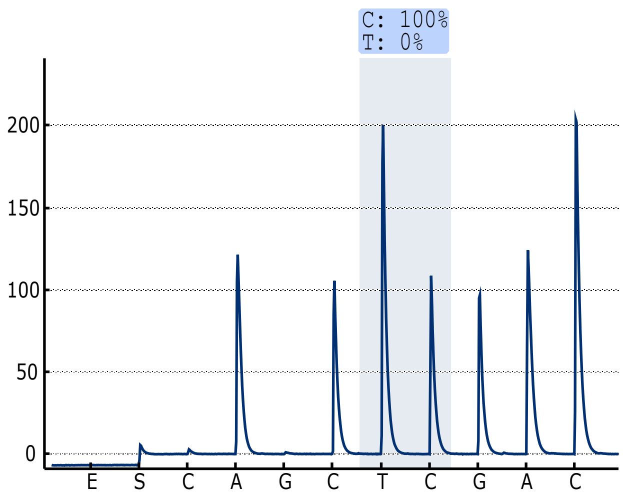

Sample ID: UPN6 AML-blasts
Assay: NRAS_Q61K

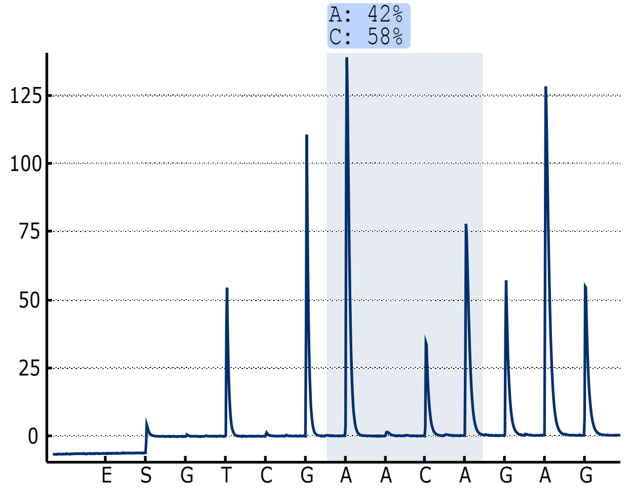

Sample ID: UPN6 BMS
Assay: NRAS_Q61K

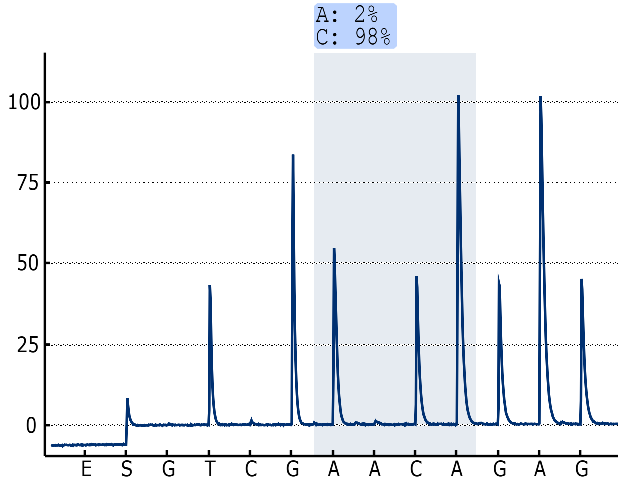

Supplement: Supplementary Figure 1 [file bcj201793x2.pdf]
